# Giant Solitary Cyst at the Site of Knee Osteoarthritis: Treatment with a Synthetic Resorbable Bone Graft Substitute and Primary Total Knee Arthroplasty

**DOI:** 10.1155/2018/1693131

**Published:** 2018-07-18

**Authors:** Andreas Thiery, Octavian Tapos, Konstantinos Anagnostakos

**Affiliations:** Zentrum für Orthopädie und Unfallchirurgie, Städtisches Klinikum Saarbrücken, Saarbrücken, Germany

## Abstract

A 48-year-old male patient presented in our department with knee osteoarthritis and a giant cystic lesion of the lateral femoral condyle. Bone biopsy of the lesion was performed. Histopathological examination confirmed the presence of a solitary bone cyst. The patient was treated by curettage of the cyst, filling with a synthetic resorbable bone graft substitute (Cerament™), and primary, cruciate-retaining total knee arthroplasty. 4 months after surgery, complete osseointegration of the bone graft substitute was evident on X-rays. The use of modern bone graft substitutes might be a novel alternative to other established techniques in the management of large bone lesions, even at the site of primary total knee replacement.

## 1. Introduction

Total knee arthroplasty is one of the most successful operations in orthopaedic surgery. However, this procedure might become demanding at the site of bone defects. Depending on the size and location of the particular osseous defect(s), various treatment modalities are available, including the use of bone cement, autologous bone grafts, structural allografts, or metallic augments [1] with or without implantation of a long-stemmed prosthesis [2].

In the past years, the use of synthetic resorbable bone graft substitutes has become a popular procedure in the management of various indications in orthopaedic and trauma surgery with good reported results [3–9]. Advantages of this treatment option involve excellent handling properties including reliable injectability, enhanced radio-opacity, and fast remodeling to bone, which might make it an attractive alternative to autologous bone or allografts [6]. Furthermore, there is no donor site morbidity [10], and it can be also performed minimal-invasive, percutaneous, or arthroscopically [4, 10].

In the present work, we would like to demonstrate an interesting case of a giant solitary bone cyst of the lateral condyle at the site of knee osteoarthritis, which was successfully treated by cyst curettage, filling with a synthetic resorbable bone graft substitute, and primary, cruciate-retaining total knee arthroplasty.

## 2. Case Report

In May 2017, a 48-year-old male patient presented in our department with pain in his right knee. The complaints were progressive over the past years. The patient denied any history of trauma. The patient did not have any comorbidities.

**Figure 1:**
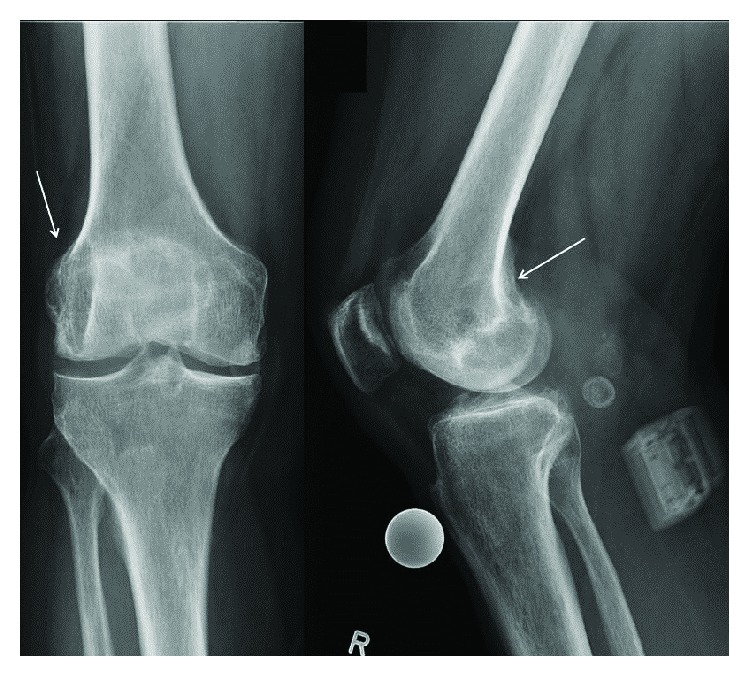
Preoperative anteroposterior and lateral radiographs of the knee demonstrated a mild osteoarthritis with a large cystic lesion of the lateral femoral condyle.

The physical examination showed a diffuse pain over the medial and lateral joint space, respectively. The Zohlen sign was positive. The collateral and cruciate ligaments were stable. The range of motion was extension/flexion 0–5–100°. Anteroposterior and lateral radiographs of the knee demonstrated a mild osteoarthritis (grade II according to Kellgren and Lawrence) with a large cystic lesion of the lateral femoral condyle (Figure 1).

**Figure 2:**
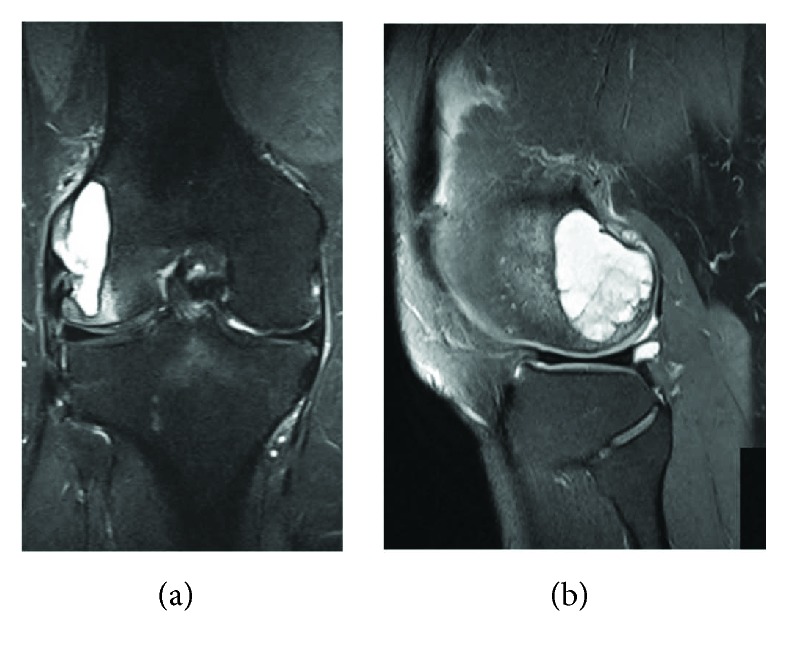
T2-weighted coronar (a) and sagittal (b) MR images of the right knee showing a giant cystic lesion of the lateral femoral condyle.

For further differential diagnosis, magnetic resonance imaging (MRI) was performed. MRI demonstrated a cystic lesion of a size of 4.2 × 3.1 × 1.2 cm with hypointensity on T1-weighted and hyperintensity on T2-weighted images (Figure 2). Diffuse cartilage lesions grade II-III according to Outerbridge of the medial compartment were evident. To exclude other pathologies, an open biopsy of the region was carried out. Histopathological examination showed the presence of a simple, solitary bone cyst without any signs of malignancy or rheumatic disease.

**Figure 3:**
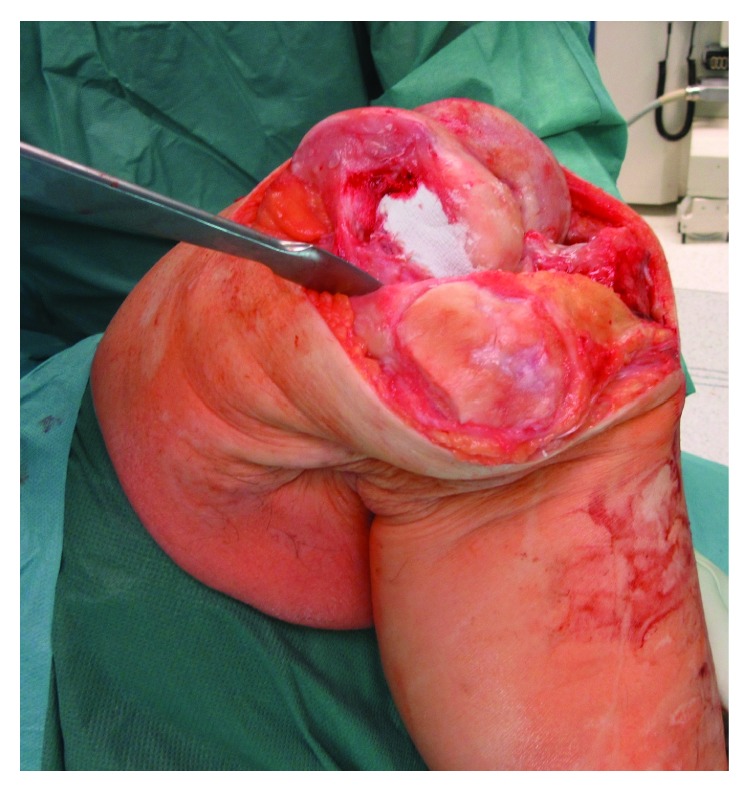
After curettage of the bone cyst, 15 ml of Cerament was inserted to augment the lateral condyle.

**Figure 4:**
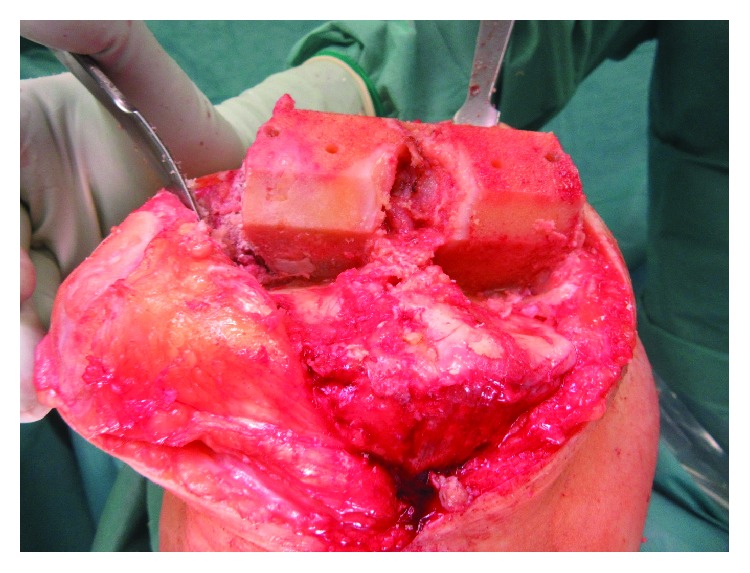
The augmented bone area of the lateral condyle became evident after the distal dorsal cutting of the femur.

**Figure 5:**
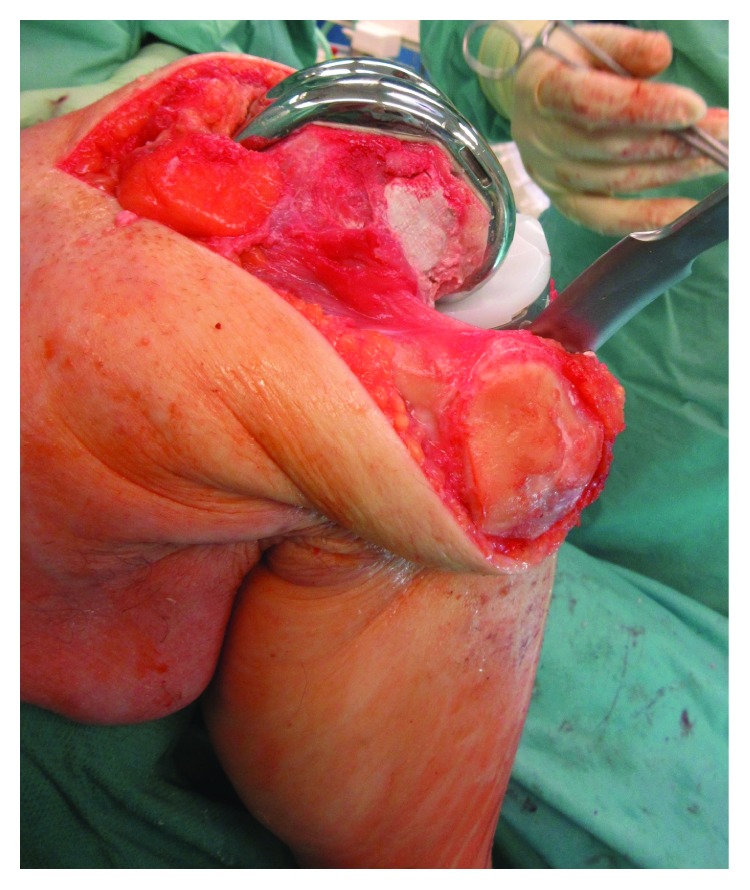
Lateral view of the operative situs after implantation of the knee endoprosthesis.

Based on the clinical, radiological, and MRI findings and the progressive complaints of the patient, the possible treatment modalities (sole filling of the cyst versus medial partial knee replacement and cyst filling versus total knee replacement and cyst filling) were discussed with the patient, and he was advised to undergo total knee replacement. Regarding the treatment of the bone cyst, the decision was made for complete curettage of the lesion and filling with a synthetic resorbable bone graft substitute (Cerament, Fa. Bonesupport, Lund, Sweden). Intraoperatively, 15 ml of Cerament were necessary to augment the cyst (Figure 3). We decided to insert the bioabsorbable bone graft prior to the cutting preparation and not vice versa, because preoperative templating of the prosthesis could not predict the amount of the bone defect that would become evident after the cutting, and we were not sure whether technical difficulties might occur regarding the creation of a smooth surface for the anchorage of the prosthesis. No synovitis or fibrinoid degeneration were intraoperatively evident. During preparation for the total knee arthroplasty and the distal, dorsal cutting of the femur, the augmented bone area of the lateral condyle was evident (Figure 4). Since only the dorsal part of the lateral condyle was evident and the remaining bone quality was good, we decided to perform a standard, cruciate-retaining total knee replacement (Triathlon® CR, Fa. Stryker, Duisburg, Germany) (Figure 5). At the end of the surgery, the intraoperative range of motion was extension/flexion 0–0–140°.

**Figure 6:**
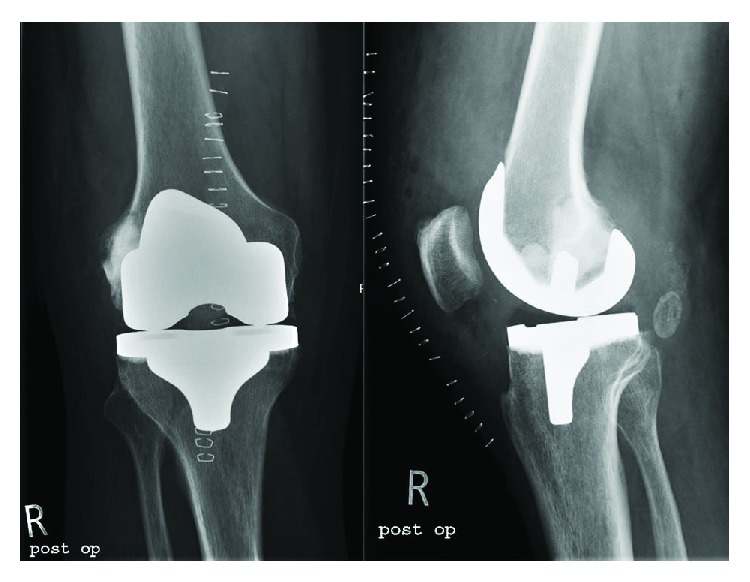
Postoperative radiographs of the right knee confirmed the correct position of the prosthesis and a proper filling of the large bone cyst with Cerament.

Postoperatively, the patient was allowed to put full weight-bearing on crutches. The further postoperative course was uneventful, and the patient was dismissed after 9 days. Postoperative radiographs of the right knee confirmed the correct position of the prosthesis and a proper filling of the large bone cyst with Cerament (Figure 6).

4 months later, the patient presented again in our department with clinical signs of an arthrofibrosis. He reported on a decrease of the range of motion after the 4th postoperative week. At presentation, the range of motion was limited to extension/flexion 0–30–85°. There were no clinical signs of an infection. Laboratory examination showed a C-reactive protein concentration of <2 mg/dL and a white cell blood count of 12,800 × 10^6^/*μ*L.

**Figure 7:**
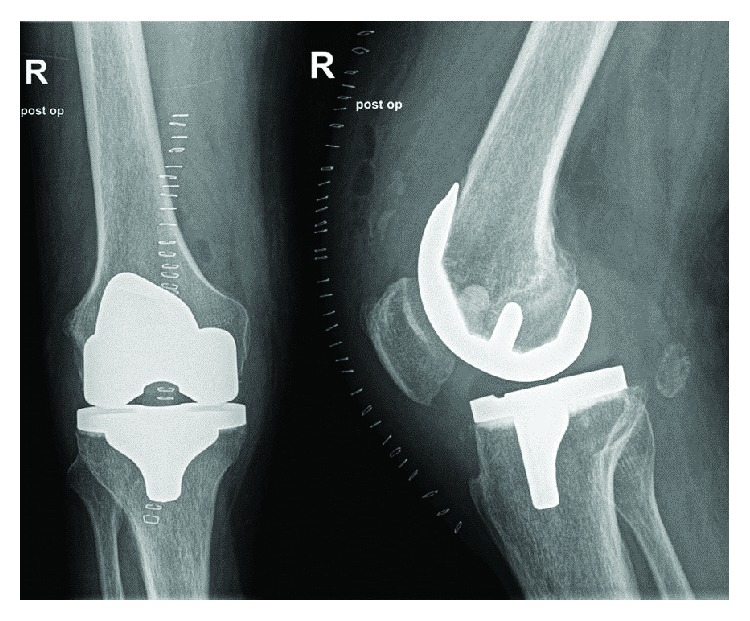
Excellent osseointegration of Cerament 4 months after filling of the giant bone cyst.

Revision surgery and an open arthrolysis were then performed. Intraoperatively, there were no signs of an infection or a third-body wear reaction due to Cerament. Multiple samples of soft tissues were taken and sent for further microbiological and histopathological examination. All microbiological findings were negative. The histopathological examination confirmed the presence of an arthrofibrosis grade 2 with >20 fibroblasts/high-power field with no evidence of a third-body wear or granulocytes. Postoperatively, continuous passive motion therapy was immediately started at the site of a femoral nerve block during the first 5 postoperative days and under full weight-bearing of the operated extremity. The further course was uneventful. Postoperative radiographs of the knee showed an excellent osseointegration of Cerament (Figure 7). At dismissal, the range of motion was extension/flexion 0–0–100° and remained during the follow-up of 12 months.

Informed consent was obtained from the patient.

## 3. Discussion

To the best of our knowledge, the present article is the first one to report on the successful treatment of a giant intraosseous solitary cyst at the site of knee osteoarthritis by cyst curettage, filling with a synthetic resorbable bone graft substitute, and implantation of a primary, cruciate-retaining total knee arthroplasty. The rapid bone remodeling on plain radiographs indicates that this method might be a good alternative to other surgical techniques.

Although intraosseous cystic lesions are common in patients with advanced knee osteoarthritis, literature data about the coexistence of a giant intraosseous cyst, known as geode, and knee osteoarthritis are scarce. We were able to identify only a single report in English literature [11]. Ohishi et al. described such a case in an 83-year-old male patient with a cyst in the medial condyle with a size of 3.5 × 3.5 × 1.6 cm [11]. A medial femoral cortical defect and a small bone break on the articular surface of the medial femoral condyle were observed. The patient was treated by total knee replacement with a long-stemmed implant. The bone cyst was curetted and packed with bone chips obtained from the lateral femoral condyle and tibial plateau. 2 years after surgery, the authors stated that the grafted bone was well incorporated with radiological loosening of the prosthesis.

The decision about the ideal treatment of such cases depends mostly on the size and localization of the bone cyst as well as the joint and leg axis deformity caused by the osteoarthritis. In our case, the fact that the augmented area became evident only after the distal posterior cutting of the femur and therefore the “ratio” augmented area/remaining natural bone was low leads us to the decision to use a standard, cruciate-retaining prosthesis with no necessity for long stems or other augmentation techniques. Moreover, we also relied on the primary stability provided by Cerament, and we did not worry about a potential fracture of the condyle or instability of the prosthesis after mobilization of the patient. In contrast to our case, the case described by Ohishi et al. [11] had an affection of the cortical bone of the medial femur, which can explain the difference in the prosthesis used.

Already 4 months after surgery, we were able to observe an excellent remodeling of the incorporated synthetic bone graft by disappearance of the cement and replacement by trabecular bone. To our opinion, the radio-opacity of Cerament allows for an easy and reliable evaluation of bone remodeling. In the case of Ohishi et al. [11], the exact remodeling cannot be sufficiently evaluated despite the statement of the authors, because, to our opinion, this cannot be measured on anteroposterior and lateral plain radiographs due to the femoral trail box of the prosthesis.

Cerament is composed of 60% *w*/*w* fast-resorbing calcium sulphate, which is intended to be quickly replaced by the newly formed bone, and of 40% *w*/*w* calcium hydroxyapatite, which acts as a long-lasting scaffold to allow further bone ingrowth [3]. Cerament triggers an endogenous precipitation of hydroxyapatite on its surface which prevents passive resorption. The sulphate dehydrate part of the implant is gradually resorbed during 7-8 weeks and being replaced by ingrowing bone that remodels to form trabecular bone, supplied by the remaining hydroxyapatite nanoparticles thus incorporated into the newly formed trabecular bone [5]. The ratio results in a mixture with compressive strength (wet) of 5–8 MPa, comparable to trabecular bone [5]. Good and excellent results have been published about its use in the management of reverse Hill-Sachs lesions [4] and tibial plateau fractures [5] and in spine [8–10], tumor [6], and hand surgery [7]. Osseointegration was not disrupted when it was used as a coating on implants [12]. As far as we know, the present case is the first one to report on the use of Cerament at the site of a total knee arthroplasty.

The postoperative course of our patient was complicated by the emergence of arthrofibrosis, which had to be treated by surgical revision. Arthrofibrosis is a known complication after total knee arthroplasty [13]. In our case, the histopathological examination of the tissue samples revealed no evidence of a third-body wear or granulocytes. Therefore, we can assume that this complication was not directly associated with the use of Cerament, and might even have happened, if we had used another surgical technique to fill the bone cyst.

In conclusion, the use of a synthetic resorbable bone graft substitute to augment a bone defect in total knee replacement might pose a good alternative to other established options such as bone cement, autologous or structural bone grafts, or metallic augments. Since no clear guidelines exist about the ideal treatment of such bone defects at the site of total knee replacement, the necessity of evolving new techniques and discussing of them is apparent. Future studies are welcome to verify the long-term follow-up of patients in which this synthetic bone graft substitute has been used.
